# Synthesis and Characterization of Multifunctional Two-Component Waterborne Polyurethane Coatings: Fluorescence, Thermostability and Flame Retardancy

**DOI:** 10.3390/polym9100492

**Published:** 2017-10-08

**Authors:** Xuan Yin, Xiaoyu Li, Yunjun Luo

**Affiliations:** School of Materials Science and Engineering, Beijing Institute of Technology, Beijing 100081, China; 3120140444@bit.edu.cn (X.Y.); xiaoyuli@bit.edu.cn (X.L.)

**Keywords:** two-component waterborne polyurethane, fluorescence, flame retardancy, coatings, TGA-FTIR

## Abstract

Fluorescent and flame-retardant two-component waterborne polyurethane coatings were synthesized using 1,5-dihydroxy naphthalene, a halogen-free polyphosphate and a hydrophilic curing agent, and their properties were systematically characterized. The average particle sizes and zeta potential values were below 170 nm and −30 mV. Meanwhile, the multifunctional two-component waterborne polyurethane coatings had strong fluorescence intensities. When comparing with the coatings with 0.5 wt % 1,5-dihydroxy naphthalene, the coatings with 1.0 wt % 1,5-dihydroxy naphthalene had a stronger microphase separation. Interestingly, the thermostability of the multifunctional coatings was remarkably improved through 1.0 wt % 1,5-dihydroxy naphthalene, and besides it belonged to nonflammable materials. Additionally, all of the coating films passed the solvent resistance testing. These samples with different amounts of 1,5-dihydroxy naphthalene are environmental friendly, especially applications that require transparent and fluorescent coatings.

## 1. Introduction

Two-component waterborne polyurethanes are important in coatings, adhesives, wood products, and automobiles because of their non-toxic, excellent properties, and an outstanding weatherability [1,2,3]. Two-component waterborne polyurethanes consist of a hydroxyl component and an isocyanate component, and are combustible materials if they are not modified with flame-retardant treatments. Although traditional two-component waterborne polyurethane coatings present a potential fire hazard, they can be functionalized using physical or chemical methods [4,5,6]. Unfortunately, few studies have reported multifunctional two-component waterborne coatings, e.g., coatings that combine fluorescence and flame retardancy properties, which have significant potential for marking and identification in polymer, chemical engineering, and coatings. Therefore, investigations on multifunctional two-component waterborne polyurethane coatings are important to produce coatings with flame-retardant and fluorescent modifications.

Halogen-free polyphosphate (OP550) is a type of phosphorus-nitrogen-based flame retardant that can coordinate flame-retardant units into backbones or branches of polyurethane chains [7]. Generally, phosphorus-nitrogen-based flame retardants are non-toxic and effective, and they form a non-volatile, protective film composed of both phosphoric and polyphosphoric acids when burned [8,9]. Additionally, flame retardants containing phosphorus and nitrogen emit various nonflammable gases to isolate combustible gases when the matrix materials are heated [10,11]. 1,5-dihydroxy naphthalene acts as fluorescent modifier and can cheaply imbue two-component waterborne polyurethanes with fluorescent properties. 1,5-dihydroxy naphthalene easily reacts with diisocyanate via traditional method because it has two hydroxyl groups in each unit.

In this paper, we synthesized a series of two-component waterborne polyurethane coatings and their films using OP550, 1,5-dihydroxy naphthalene and hydrophilic curing agent. OP550 and 1,5-dihydroxy naphthalene were covalently bonded to polyurethane chains and reacted with hydrophilic curing agent to synthesize multifunctional two-component waterborne polyurethane coatings. The multifunctional two-component waterborne polyurethane coatings showed high emulsion stability, good flame retardancy, outstanding fluorescence, and excellent thermal stability. These synthesized, multifunctional coatings were well dispersed and environmental friendly. Additionally, the coatings could be used in expanded applications, such as special coatings and for textiles requiring large area protection from radiation marks.

## 2. Materials and Methods

### 2.1. Materials

PPG1000 ($\bar{M_{n}}=1000$, industrial grade, Dawson International Inc., Buffalo Grove, IL, USA) was dried under a vacuum at 90 °C for 6 h; isophorone diisocyanate (IPDI, analytical grade, Aladdin); dimethylol propionic acid (DMPA, chemically pure, Aladdin) was dried under vacuum at 90 °C for 6 h; 1,5-dihydroxy naphthalene (DN, 99%, Aladdin) was dried under vacuum at 80 °C for 12 h; halogen free poly phosphate (OP550, $\bar{M_{n}}=831$, industrial grade, Clariant AG, Muttenz, Switzerland) was dried under vacuum at 90 °C for 4 h; acetone (chemically pure, Beijing Chemical Reagents Company, Beijing, China) was dried over a 4 Å molecular sieve (Beijing Pengcai Chemical Reagent Co., Ltd., Beijing, China) for 72 h; triethylamine (TEA, chemically pure, Beijing Yili Fine Chemicals Co., Ltd., Beijing, China) was soaked by 4 Å molecular sieve for 72 h; hydrophilic curing agent (M12A4, Lab homemade [12]); antifoaming agent (BYK 001, chemically pure, BYK Additives & Instruments, Wesel, Germany) was dried under vacuum at 80 °C for 4 h; wetting agent (BYK348, chemically pure, BYK Additives & Instruments, Wesel, Germany) was dried under vacuum at 80 °C for 4 h; flow control agent (BYK333, chemically pure, BYK Additives & Instruments, Wesel, Germany) was dried under vacuum at 80 °C for 4 h.

### 2.2. Methods

PPG1000, OP550 (the weight ratio of OP550 to total mass of raw materials was 15 wt %), and IPDI were added into 250 mL four-neck flask with a condenser pipe at 80 °C for 2 h. Then, 5.5 wt % DMPA and 1,5-dihydroxy naphthalene with some acetone were added for 4 to 5 h, and then cooled to 40 °C. Next, 5.5 wt % TEA was added to the flask at 40 °C for 30 min before adding deionized water (solid content was 30%) at 3000 rpm for 30 min. The emulsion was removed with acetone at 40 °C rotary evaporation for 2 h. The stoichiometric ratio between –NCO and –OH was fixed 0.9. Then, the hydrophilic curing agent was added (the stoichiometric ratio was 1.6) into the emulsion with antifoaming agent, wetting agent and flow control agent, which was under ultrasonic vibration for 30 min to wait for complete reaction. The resultant emulsions were multifunctional two-component waterborne polyurethanes with fluorescence and flame retardancy (NOWPUs). Six different NOWPU samples were prepared, with various 1,5-dihydroxy naphthalene content of 0 (for control experiment) 0.1, 0.5, 1.0, 1.5, and 2.0 wt %. These samples were named as NOWPU-X, with “X” representing the DN content. The NOWPUs was dropped into a Teflon plate to prepare films at 60 °C for 48 h on vacuum drying oven. The formulation is listed in Table 1.

### 2.3. Characterizations

The NOWPUs was dropped into a Teflon plate at 60 °C for 48 h on vacuum drying oven (DZF-6050, Nanjing, China). Then, the films were dried in a vacuum system (DZF-6050, Nanjing, China) at 80 °C for 12 h.

#### 2.3.1. NOWPU Emulsion

Grain size, zeta potential and viscosity. The grain size and zeta potential of NOWPUs were tested at 25 °C with Nanosizer (Malvern zetasizer Nano ZS90, Malvern Instruments Ltd., Malvern, UK). The viscosity of NOWPUs were tested by Brookfield programmable DV-II + Pro Viscometer (Brookfield Engineering Labs. Inc., Boston, MA, USA).

Anti-ultraviolet and Fluorescence spectrum. The U-3010 Hitachi ultraviolet-visible (UV-vis) spectrophotometer (Hitachi Ltd., Tokyo, Japan) was used to test 1,5-dihydroxy naphthalene and NOWPUs. The F-2500 Hitachi fluorescence spectrophotometer (Hitachi Ltd., Tokyo, Japan) was used to test 1,5-dihydroxy naphthalene and NOWPUs.

#### 2.3.2. NOWPU Coatings and Films

Fourier transform infrared spectroscopy. The FTIR spectra were recorded on Nicolet 8700 Fourier transformed infrared spectroscopy (FTIR, Thermo Nicolet Corporation, Waltham, MA, USA). The samples were dried at 80 °C for 24 h.

Differential scanning calorimeter. The glass-transition temperatures of NOWPU films were tested by differential scanning calorimeter (Switzerland Mettler Toledo DSC 1 differential scanning calorimeter, Mettler-Toledo International Inc., Zurich, Switzerland). The measurements were tested at liquid nitrogen condition from −100 to 150 °C at a warming speed 10 °C/min. The samples were dried at 80 °C for 24 h.

Thermogravimetric analysis. The thermal degeneration NOWPU films and char yield of NOWPU coatings were tested by thermogravimetric analysis (Switzerland Mettler TGA/DSC differential thermal scanners, Mettler-Toledo International Inc., Zurich, Switzerland). The measurements were tested at nitrogen condition from 30 to 500 °C at a warming speed 10 °C/min. The samples were dried at 80 °C for 24 h.

TGA-FTIR analysis. The gaseous product of thermal degeneration of NOWPU coatings were tested by thermogravimetric infrared analysis (Mettler Toledo TGA/DSC1-Ncolet 6700 FTIR, Mettler-Toledo International Inc., Zurich, Switzerland). The measurements were tested at a nitrogen condition from 30 to 500 °C at a warming speed 10 °C/min. The samples were dried at 80 °C for 24 h.

Fourier transform infrared spectroscopy of char residue. The NOWPU films were set in a tube furnace at nitrogen condition. The procedure of the tube furnace was set at 100 °C, 200 °C, 300 °C, 350 °C, 450 °C, and 500 °C for 10 min for each stage. Next, the residue was tested through FTIR. The residue was mixed with dried KBr and then pressed into tablets.

Limiting oxygen index. The limiting oxygen index (LOI) of NOWPUs was determined using the digital display oxygen index apparatus LFY-606B (Shandong textile science and technology, Jinan, China) according to the combustion performance of the oxygen index standard test ASTM D2863-77 by LFY-606B digital display oxygen index apparatus (Shandong Academy of Sciences, Jinan, China). The NOWPU films were cut to 150 mm × 50 mm splines (each sample had five strips).

UL-94 Vertical burning test. UL-94 of NOWPU films were conducted by a CZF-II horizontal and vertical burning tester (Jiang Ning Analysis Instrument Co., Nanjing, China). The specimens used were 150 mm × 50 mm × 2 mm according to the UL-94 tests (ASTM D3801-1996 standard).

Scanning electron microscopy. SEM (Hitachi S-4800 field-emission scanning electron microscopy, Hitachi, Co., Tokyo, Japan) was used to investigate the surface of char residues of the NOWPU films at 500 °C in a tube furnace. The scanned region was approximately 100 mm × 50 mm.

Application properties. The application properties of NOWPU coatings were tested according relative Chinese standard. The pencil hardness of NOWPU coatings was tested according GB/T 6739-1996. The solvent resistance of coatings was the test through wiping method. We used a hollow tubular container with tip of stringy. The tip of the stringy dipped acetone solvent. The films were wiped one hundred to and fro at a second time. The films that did not appeared grinning, dulling and through dissolved mean passing solvent resistance measurement. For pencil hardness and solvent resistance measurements of NOWPU coating films, the sample preparation referred to GB1727-92. The light transmissivity of NOWPU coatings were tested by UV-vis spectra (F-2500 Hitachi fluorescence spectrophotometer) and Ref101N-min 60° glossmeter (Sheen Instruments, Surrey, UK). The NOWPU films were spun on a 25.6 mm × 72.4 mm × 2 mm glass slide and then dried in a vacuum drying oven at 80 °C for 12 h.

## 3. Results

### 3.1. Structure Analysis of the NOWPU Films

**Figure 1:**
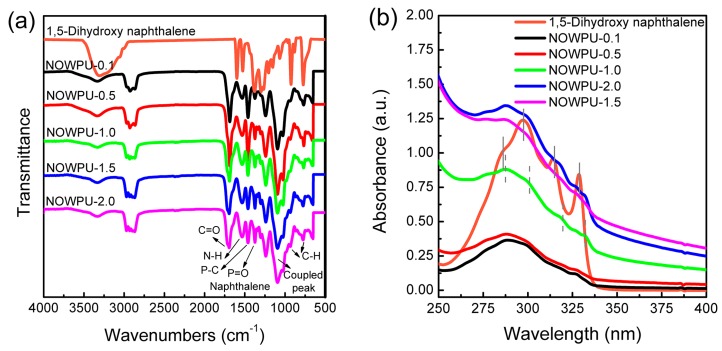
(**a**) Fourier transformed infrared spectroscopy (FTIR) and (**b**) UV-vis spectrum of NOWPUs.

In the FTIR spectra (Figure 1a), the absorption of C=O at 1694 cm^−1^ and that of N–H at 1533 cm^−1^ came from the main polyurethane chain. The absorptions of P–C and P=O derived from OP550 unit was at 1459 cm^−1^ and 1238 cm^−1^, respectively. The coupled absorption of $–{\mathrm{SO}}_{3}^{-}$, C–N, C–O, and N–CO–O at 1120 cm^−1^ was due to add hydrophilic curing agent. Additionally, the absorption of naphthalene at 1459 cm^−1^ (with P–C overlapping) and the absorptions of C–H at 865 cm^−1^ and 779 cm^−1^ were according to 1,5-dihydroxy naphthalene. When compared with the spectrum of 1,5-dihydroxy naphthalene, the spectra of the NOWPUs showed a bathochromic shift in the absorption maxima from 297 to 305 nm (Figure 1b).

### 3.2. Particle Size, Viscosity, and Emulsion Stability

**Figure 2:**
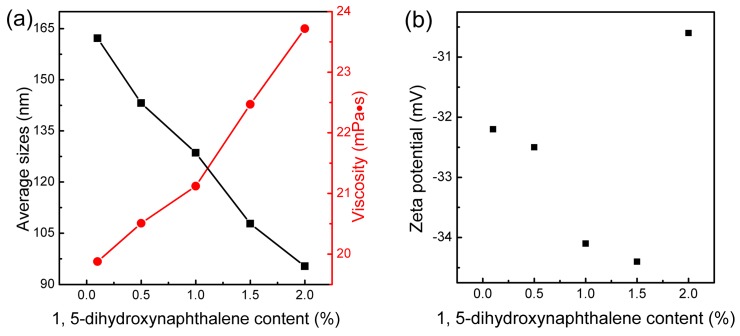
(**a**) Emulsion particle size, viscosity; and, (**b**) zeta potential of NOWPUs.

The average grain sizes of the NOWPUs increased from 96.7 to 165.0 nm upon the addition of 1,5-dihydroxy naphthalene (Figure 2a). Meanwhile, the viscosity decreased from 23.6 to 19.8 mPa·s (Figure 2a) as the 1,5-dihydroxy naphthalene content increased. The zeta potential values for the NOWPUs were all lower than −30 mV (Figure 2b).

### 3.3. Fluorescence

#### 3.3.1. Stereohindrance Factors

**Figure 3:**
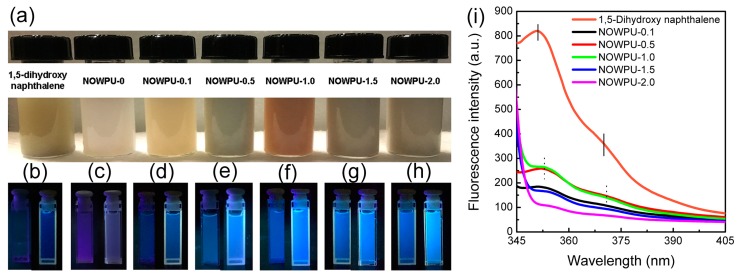
(**a**) Photographs of NOWPUs by natural; and (**b**–**h**) UV light; and (**i**) fluorescence emission spectrum of NOWPUs: the left of (**b**–**h**) was radiated at 254 nm UV lamp, the right of (**b**–**h**) was radiated at 365 nm UV lamp (The samples for natural light were original emulsion, concentration of NOWPUs for UV lamp = 10^−3^ g/mL in water).

Figure 3a–h show photographs of the 1,5-dihydroxy naphthalene and the NOWPUs in water under room light and UV light, respectively. The NOWPUs displayed a fairly strong fluorescence emission (Figure 3i), which had a weak bathochromic shift of approximately 4 nm (from 351 to 355 nm) in the emission maximum.

#### 3.3.2. Temperature Influence

**Figure 4:**
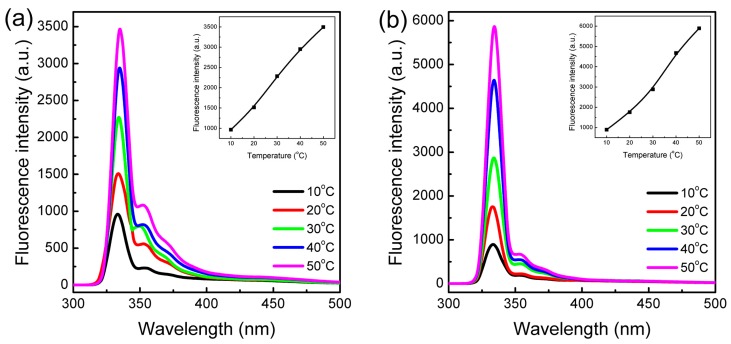
Fluorescence spectrum of (**a**) NOWPU-0.5 and (**b**) NOWPU-1.0 with various temperatures (concentration of NOWPUs = 10^−3^ g/mL in water, λ_ex_ of NOWPU-0.5 = 331 nm, λ_ex_ of NOWPU-1.0 = 332 nm).

Figure 4 shows the fluorescence spectra of NOWPU-0.5 and NOWPU-1.0 at various temperatures. The fluorescence intensity remarkably increased as the temperature increased, especially for NOWPU-1.0.

#### 3.3.3. Solvatochromic Factor

**Figure 5:**
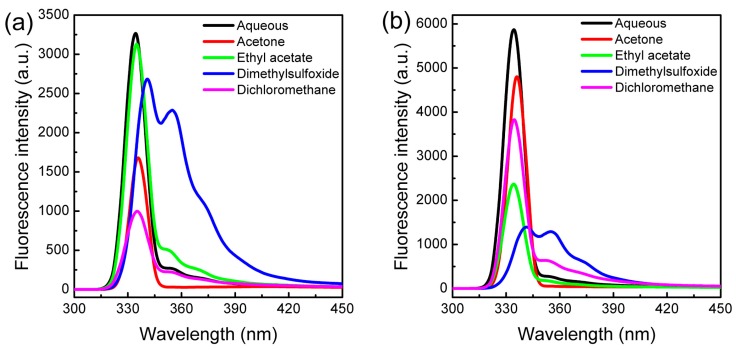
Fluorescence spectrum of (**a**) NOWPU-0.5 and (**b**) NOWPU-1.0 with various solvents (Concentration of NOWPUs = 10^−3^ g/mL in water, λ_ex_ of NOWPU-0.5 = 331 nm, λ_ex_ of NOWPU-1.0 = 332 nm).

As shown in Figure 5a, the excitation maximum in NOWPU-0.5 had a bathochromic shift from 334 to 341 nm (peak value) as the solvent polarity decreased. Similarly, the excitation maximum of NOWPU-1.0 had a bathochromic shift from 334 to 342 nm as the solvent polarity decreased (Figure 5b).

### 3.4. Thermal Properties and Mechanism

#### 3.4.1. Thermal Properties Determined by DSC (Differential Scanning Calorimetry)

**Figure 6:**
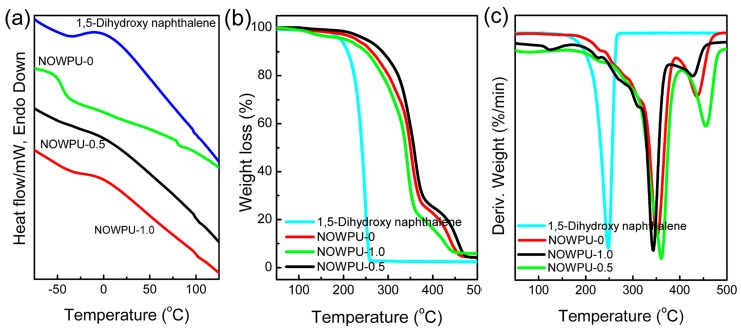
(**a**) DSC (differential scanning calorimetry) curves; (**b**) TGA (thermogravimetric analysis) curves and (**c**) DTG (differential thermal gravity) curves of NOWPU films.

The glass-transition temperature of the soft segment (*T_gs_*) remarkably decreased and the glass-transition temperature of the hard segment (*T_gh_*) slightly increased, which increased the difference between *T_gs_* and *T_gh_* (*ΔT_g_*) and caused a strong microphase separation. The microphase separation of NOWPU-0.5 and NOWPU-1.0 was larger than that of the NOWPU-0 (Figure 6a and Table 2).

#### 3.4.2. Thermal Properties Determined by TGA (Thermogravimetric Analysis)

The maximum decomposition temperature (*T_max_*) was 231 °C with 1.0 wt % 1,5-dihydroxy naphthalene in the first stage (Figure 6b,c and Table 3). The maximum decomposition speed (*V_max_*) was 0.0008 °C/min (the lowest value in the NOWPU films). In the second stage, the maximum *T*_0_ was 252 °C with 1.0 wt % 1,5-dihydroxy naphthalene. Meanwhile, the maximum *T_max_* and *V_max_* were 324 °C and 0.0072 °C/min, respectively. Similarly, NOWPU-1.0 had the maximum *T*_0_ and *T_max_* in the third stage. Ultimately, the maximum initial decomposition temperature (*T*_0_), *T_max_*, and *V_max_* values were 409 °C, 456 °C, and 0.0047 °C/min, respectively, in the end stage.

#### 3.4.3. Thermal Mechanism

**Figure 7:**
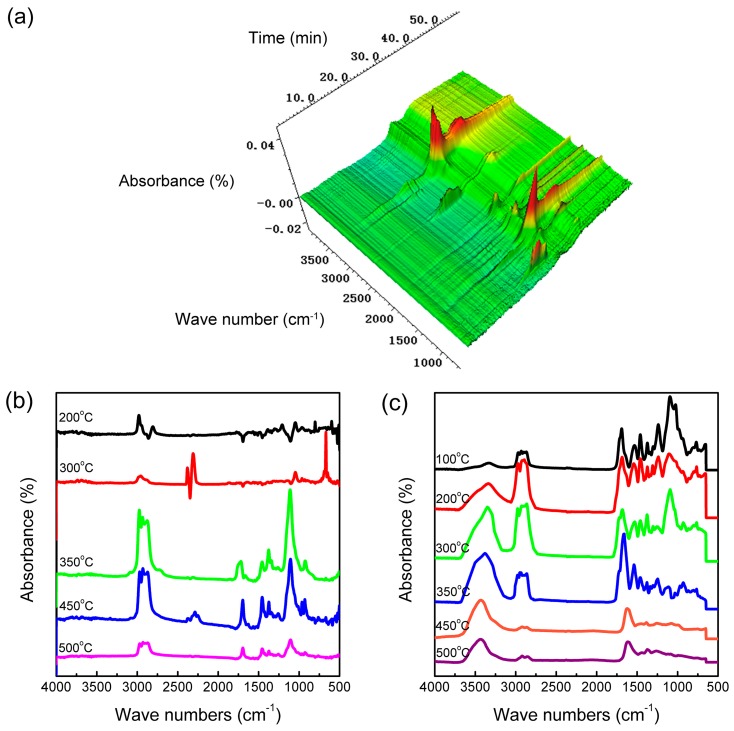
TGA-FTIR and FTIR of carbon residue of NOWPU-1.0 film: (**a**) three-dimensional spectrum; (**b**) TGA-FTIR at various temperature; and, (**c**) FTIR of carbon residue at various temperature.

NOWPU-1.0 was used to analyze the TGA-FTIR and FTIR spectra of the films at various decomposition temperatures (Figure 7). Figure 7a is a 3D image from the TGA-FTIR tests, and Figure 7b is the FTIR spectrum obtained from the TGA-FTIR (Figure 7a). Figure 7b shows the very weak characteristic absorption peaks of CO_2_, –NCO, and HCN at 2379, 2357, and 2315 cm^−1^, respectively, at 200 °C. At 300 °C, the absorptions of P–OH and SO_2_ were observed at 1708 and 1046 cm^−1^, respectively. Meanwhile, the stretching vibrations of the C–H in the benzene ring were at 668 and 650 cm^−1^ in the fingerprint region. Furthermore, NH_3_ (925 cm^−1^) was present at this stage. As the temperature continued to increase, the absorption peaks of C=O, –CHO, and the ester bond were at 1718, 2720, and 1111 cm^−1^, respectively. Meanwhile, NH_3_ (925 cm^−1^) was also created at this stage. The absorption peaks of methyl or methylene groups and ether bonds were still occurring. At 450 °C, the absorption peak of the ether bond at 1105 cm^−1^ and the characteristic absorption peak of C=O at 1696 cm^−1^ increased in intensity. NH_3_ was still present at 929 cm^−1^.

**Scheme 1:**
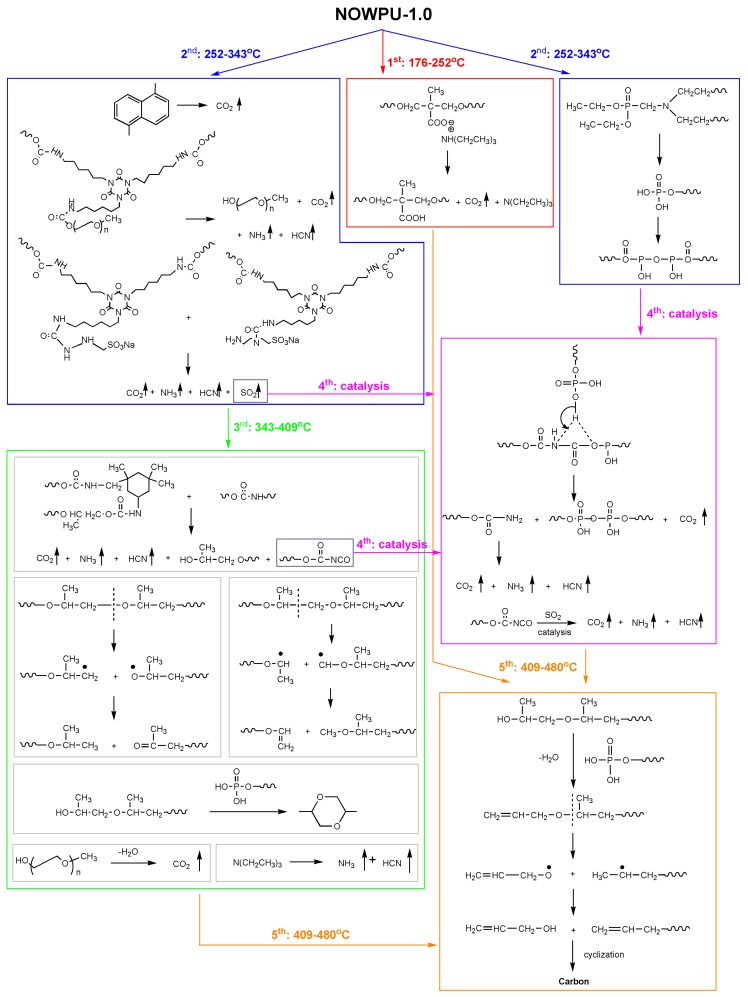
Thermal degradation mechanism mode of NOWPU-1.0 coatings.

Figure 7c shows the decomposition of OP550 and the neutral segments. The absorption peaks of CO_2_ and SO_2_ were 2379 and 1046 cm^−1^, respectively, at 200 °C. As the temperature continued to increase, an important absorption peak for P–OH appeared at 1708 cm^−1^. Meanwhile, the stretching vibration peaks of carbamate were seen at 1740 cm^−1^. Furthermore, the stretching vibrations of the C–H in the benzene ring were at 668 and 650 cm^−1^ in the fingerprint region. At 350 °C, the absorption peak for –CHO at 2720 cm^−1^ was seen. Meanwhile, the carbonyl peak was at 1718 cm^−1^, and the ether bond peak was at 1111 cm^−1^. Additionally, the NH_3_ peak appeared (925 cm^−1^). Further, at 450 °C, a stronger carbon peak was observed at 1696 cm^−1^. When the temperature was 500 °C, no peaks were observed in the FTIR and TGA-FTIR spectra. The specific thermal decomposition mechanism was as follows (Scheme 1).

### 3.5. Flame Retardancy and Mechanism

**Figure 8:**
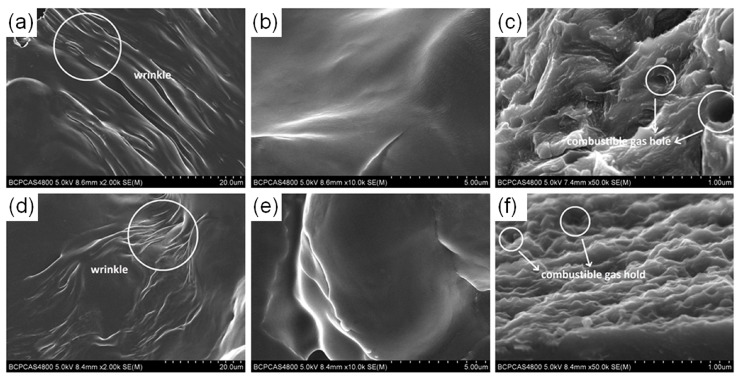
SEM images of (**a**–**c**) NOWPU-0.5 and (**d**–**f**) NOWPU-1.0.

Table 4 shows that the NOWPU-1.0 and NOWPU-0.5 films are nonflammable materials, and the oxygen limit index of NOWPU-1.0 reached 31.2%. Meanwhile, the vertical burning results for the two films reached the V-0 level. The cladding layer (wrinkle) appeared on the surface of the carbon residues (Figure 8a,d). Figure 8b,e show that the surface of the char residue from NOWPU-1.0 was thicker and smoother than that from NOWPU-0.5. In Figure 8c,f, the excited combustible gas was held inside the carbon layers, and the carbon layers effectively prevented the combustible gas from reaching the interior. Meanwhile, NOWPU-1.0 had smaller holes on its surface than NOWPU-0.5.

### 3.6. Application Evaluation

**Figure 9:**
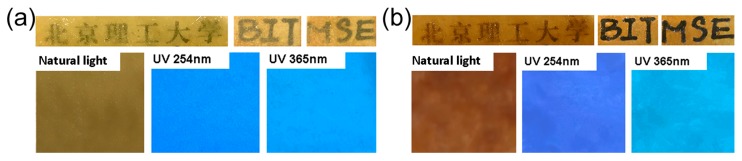
Coating films radiated by UV and natural lights: (**a**) NOWPU-0.5; (**b**) NOWPU-1.0.

The evaluation parameters for coating applications are listed in Table 5. The pencil hardness of the NOWPU films increased after adding the rigid 1,5-dihydroxy naphthalene. All of the films passed the solvent resistance testing with the addition of a rigid organic compound. The NOWPU-1.0 had a high transmittance for natural and visible lights, 100.0% and 95.5%, respectively. Meanwhile, NOWPU-1.0 had an extremely low transmittance of UV light at 200 nm. Figure 9 shows the films radiated through the UV and natural lights. NOWPU-1.0 had a wider color range for the different UV radiations, and the word below the NOWPU-1.0 coating film was more distinct than that below the NOWPU-0.5 film, except for the darker film color.

## 4. Discussion

The multifunctional two-component waterborne polyurethanes were synthesized via acetone method and chemical crosslinking. UV spectra shows the absorption bands associated with the naphthalene unit, which further confirmed that the 1,5-dihydroxy naphthalene successfully attached to the waterborne polyurethane chain. When compared with the spectrum of 1,5-dihydroxy naphthalene, the spectra of the NOWPUs showed a bathochromic shift in the absorption maxima from 297 to 305 nm, which was a result of the interactions between the dipole molecules and ions in the NOWPU because the transfer of π–π* occurred at the same time [13,14].

The variation of average grain sizes suggests that more 1,5-dihydroxy naphthalene increased the blocking ability and volume of the polyurethane polyol chains, leading to more interchain entanglement and harder intramolecular rotation. As a result, the movement of polyurethane chains was restricted, causing the larger particle sizes with more 1,5-dihydroxy naphthalene. Emulsion viscosity is one of the most important performance indexes for storage stability and application properties, and the viscosity is increased by a small particle size [8]. The 1,5-dihydroxy naphthalene acted as a rigid, organic molecule that increased particle size to decrease viscosity because it weakened intermolecular force. Hence, the viscosity of the NOWPUs decreased from 23.6 to 19.8 mPa·s as the 1,5-dihydroxy naphthalene content increased. The waterborne polyurethane emulsion was stable due to the mutual repulsion caused by the electric double layers [15]. The zeta potential values for the NOWPUs, which were all lower than −30 mV, suggested sufficient colloidal stabilities [15].

Multifunctional two-component waterborne polyurethanes had strong fluorescence intensity. In fact, 1,5-dihydroxy naphthalene displayed a fairly strong fluorescence emission, which had a weak bathochromic shift of approximately 4 nm (from 351 to 355 nm) in the emission maximum in the NOWPUs. Besides, the fluorescence intensity of the NOWPUs reached a maximum with 1.0 wt % 1,5-dihydroxy naphthalene (NOWPU-1.0) [15,16]. Further, the fluorescence intensity remarkably increased as the temperature increased, especially for NOWPU-1.0 because of the reduced energy difference during the attachment of 1,5-dihydroxy naphthalene to polyurethane chains [17,18]. Therefore, the lowest excited singlet state to lowest excited triplet state transition could occur along with the conversion of the lowest excited triplet state to the lowest excited singlet state in the NOWPU excited by thermal energy.

Besides, the excitation maximum in NOWPU-0.5 had a bathochromic shift from 334 to 341 nm (peak value) as the solvent polarity decreased, which indicated that the maximum fluorescence emission was almost insensitive to the solvent polarity. Similarly, the excitation maximum of NOWPU-1.0 had a bathochromic shift from 334 to 342 nm as the solvent polarity decreased. The solvent was redirected due to the changed dipole moment [19]. The NOWPU-1.0 had a stronger microphase separation than that of NOWPU-0.5 according to the DSC results, which also influenced the bathochromic shift.

The NOWPU films had two *T_g_*: *T_gh_* and *T_gs_* [20]. Generally, the difference (*ΔT_g_*) in the *T_g_* between *T_gh_* and *T_gs_* can be utilized to characterize the microphase separation degree in polyurethanes [20]. Adding 1,5-dihydroxy naphthalene increased the hard segment proportion of the polyurethane. It enhanced the influence of the carbamate, which was created by hydroxyl and isocyanato groups on the hard segment. The *T_gs_* remarkably decreased and the *T_gh_* slightly increased, which increased the *ΔT_g_* and caused a strong microphase separation. Consequently, the movement of the two-component waterborne polyurethane chains was weakened by 1,5-dihydroxy naphthalene due to the strong intermolecular interactions and poor soft segment movement [3]. The microphase separation of NOWPU-0.5 and NOWPU-1.0 was lager than that of the NOWPU-0 because the *T_gs_* of 1,5-dihydroxy naphthalene is much larger than that of NOWPU-0. The NOWPU-1.0 had the maximal microphase separation, which influenced the most conspicuous bathochromic shift in various solvent. Although the microphase separation of the two films increased, the *ΔT_g_* value was still less than that of 1,5-dihydroxy naphthalene. Therefore, the two films were less polar than 1,5-dihydroxy naphthalene.

The first stage was the decomposition of OP550 and neutralization in the NOWPUs. The *T_max_* and *V_max_* were 231 °C and 0.0008 °C/min (the lowest value in the NOWPU films) in NOWPU-1.0. Although the *T*_0_ decreased with the addition of 1,5-dihydroxy naphthalene, the naphthalene restrained decomposition during the first stage. In the second stage, there happened the decomposition of the sulfonate in the curing agent, the organic phosphate and the naphthalene. The maximum *T*_0_, *T_max_* and *V_max_* were 252 °C, 324 °C and 0.0072 °C/min in NOWPU-1.0, respectively, which indicated the addition accelerated the decomposition because of the residual from the former stage and the decomposition of 1,5-dihydroxy naphthalene [13,17]. The third stage was the decomposition of the residual hard segment. Similarly, NOWPU-1.0 had the maximum *T*_0_ and *T_max_*. 1,5-Dihydroxy naphthalene improved the decomposition temperature and delayed the decomposition speed [21]. Ultimately, the soft segment decomposed in the end stage. The maximum *T_0_*, *T_max_*, and *V_max_* values were 409 °C, 456 °C and 0.0047 °C/min, respectively, in NOWPU-1.0. Hence, the thermostability was improved by 1,5-dihydroxy naphthalene due to the carbon layers form via the catalysis from the phosphate groups and SO_2_. A higher residual carbon yield resulted in better thermostability and flame retardancy [8,22].

NOWPU-1.0 was used to analyze the TGA-FTIR and FTIR spectra of the films at various decomposition temperatures. The very weak characteristic absorption peaks of CO_2_, –NCO, and HCN at 2379, 2357 and 2315 cm^−1^, respectively, which indicated that the decomposition of the neutralized segment and organic phosphate occurred at 200 °C. However, it just began soon. At 300 °C, the absorptions of P–OH and SO_2_ were observed at 1708 and 1046 cm^−1^, respectively. Meanwhile, the stretching vibrations of the C–H in the benzene ring were at 668 and 650 cm^−1^ in the fingerprint region. Furthermore, NH_3_ (925 cm^−1^) was present at this stage due to the decomposition of the residual chain segment. As the temperature continued to increase, the absorption peaks of C=O, –CHO and the ester bond were at 1718, 2720, and 1111 cm^−1^, respectively. Meanwhile, NH_3_ (925 cm^−1^) was also created at this stage. The absorption peaks of methyl or methylene groups and ether bonds were still occurring, which meant that the hard segment decomposed to produce micromolecular ketones, aldehydes, ethers, and inert gases [23]. At 450 °C, the absorption peak of the ether bond at 1105 cm^−1^ and the characteristic absorption peak of C=O at 1696 cm^−1^ increased in intensity. NH_3_ was still present at 929 cm^−1^. In this stage, the soft segment decomposed to create micromolecular ketones and ethers.

During the decomposition of OP550 and the neutral segments, the very weak absorption peaks of CO_2_ and SO_2_ were 2379 and 1046 cm^−1^, respectively, at 200 °C. However, it just began soon. As the temperature continued to increase, an important absorption peak for P–OH appeared at 1708 cm^−1^. Meanwhile, the stretching vibration peaks of carbamate were seen at 1740 cm^−1^. Furthermore, the stretching vibrations of the C–H in the benzene ring were at 668 and 650 cm^−1^ in fingerprint region. At 350 °C, the absorption for –CHO at 2720 cm^−1^ was seen. Meanwhile, the carbonyl peak was at 1718 cm^−1^, and the ether bond peak was at 1111 cm^−1^. The decomposition in this stage created micromolecular ketones, aldehydes, and ethers. Additionally, the NH_3_ peak appeared (925 cm^−1^), which meant the residual hard segment decomposed to produce inert gases. Further, at 450 °C, the decomposition produced isocyanate, polyether polyols, and phosphoric acid dimers, which formed polyphosphoric acid via polycondensation of the hydroxide radical. The decomposition products were consistent with the decomposition of the soft segment. A stronger carbon peak was observed at 1696 cm^−1^, which was consistent with the TGA and TGA-FTIR results at 450 °C. When the temperature was 500 °C, no peaks were observed in the FTIR and TGA-FTIR spectra, which was consistent with the TGA results. Therefore, the two-component waterborne polyurethanes completely decomposed at 500 °C.

The oxygen limit index of NOWPU-1.0 reached 31.2%, and the vertical burning results for the two films reached the V-0 level, which indicated the that two NOWPU films had good flame retardancy [16]. From the SEM images of the char residues derived from the NOWPUs, it showed that the carbon residues notably increased upon the addition of 1,5-dihydroxy naphthalene. The phosphate groups (phosphoric acid and polyphosphoric acid) of the combustion products provide a cladding layer (wrinkle) on the surface of the two-component waterborne polyurethanes [24], which controlled enough energetic growth to decrease the energy density and prevent surface heat transmission from the combustible gas in the matrix during burning [25,26]. The more subtle wrinkles indicated tighter carbon layers. Meanwhile NOWPU-1.0 had better carbon layers after decomposition than NOWPU-0.5, which meant that NOWPU-1.0 had better flame retardancy than NOWPU-0.5 [8]. Additionally, the surface of the char residue from NOWPU-1.0 was thicker and smoother than that from NOWPU-0.5, which was consist with the TGA and flame-retardant testing results. From the SEM images, they excited combustible gas was held inside the carbon layers, and the carbon layers effectively prevented the combustible gas from reaching the interior. Meanwhile, NOWPU-1.0 had smaller holes on its surface than NOWPU-0.5 because of the tighter carbon layers. Therefore, 1,5-dihydroxy naphthalene accelerated the condensed phase flame-retardant effect in the matrix and improved the flame retardancy of the two-component waterborne polyurethanes.

The pencil hardness of the NOWPU films increased after adding the rigid 1,5-dihydroxy naphthalene, which provided large backbone groups for the macromolecule chains to increase the rotational steric hindrance and improve the rigidity of the macromolecule chains to improve the pencil hardness. All of the films passed the solvent resistance testing with the addition of a rigid organic compound, because the intramolecular and intermolecular hydrogen bonds were enhanced and provided a strong interface interactions and binding forces to tighten the network structures [27]. Consequently, the crosslinking network structure acted as a barrier to reduce the intrusion points during the solvent intrusion into the interior through the coatings. The NOWPU-1.0 had a high transmittance for natural and visible lights, 100.0% and 95.5%, respectively, because the light scattering was caused by the phase separation [27]. NOWPU-1.0 had the most obvious phase separation according to the DSC results. Besides, NOWPU-1.0 had an extremely low transmittance of UV light at 200 nm, and the film could be used in specific areas for functional textiles that need to shield UV light and have a high gloss. NOWPU-1.0 had a wider color range for the different UV radiations, and the word below the NOWPU-1.0 coating film was more distinct than that below the NOWPU-0.5 film, except for the darker film color, which could introduce an application of fluorescent coatings.

## 5. Conclusions

In this paper, we reported the synthesis and properties of multifunctional two-component waterborne polyurethane coatings created using OP550, 1,5-dihydroxy naphthalene, and a hydrophilic curing agent. The average emulsion particle sizes and the zeta potential of the NOWPUs were all below 170 nm and −30 mV, respectively. The fluorescence intensities of the multifunctional two-component waterborne polyurethane coatings were strong, especially those of NOWPU-1.0. Additionally, NOWPU-1.0 had stronger microphase separation, thermostability, and flame retardancy than that of NOWPU-0.5. NOWPU-1.0 had a wide color gamut during UV radiation and a high transparency, but NOWPU-0.5 had a lighter color appearance. Therefore, multifunctional two-component waterborne polyurethanes can be applied to special coatings and textiles that require large area protection from radiation marks.

## Figures and Tables

**Table 1:** Formulation of multifunctional two-component waterborne polyurethanes with fluorescence and flame retardancy (NOWPUs).

Sample	1,5-dihydroxy Naphthalene (g)	PPG1000 (g)	OP550 (g)	IPDI (g)	DMPA (g)	TEA (g)	Acetone (g)	M12A4 (g)	Deionized Water (g)
GOWPU-1.6	0.0	50.0	13.5	20.9	5.0	5.0	20.0	7.6	238
NOWPU-0.1	0.1	50.0	14.5	21.1	5.2	5.2	20.8	7.7	242
NOWPU-0.5	1.0	50.0	15.0	22.6	5.4	5.4	21.6	8.0	251
NOWPU-1.0	2.1	50.0	15.5	24.3	5.6	5.6	22.4	8.3	259
NOWPU-1.5	3.2	50.0	16.2	26.2	5.8	5.8	23.2	8.6	270
NOWPU-2.0	5.8	50.0	16.8	28.2	6.0	6.0	24.0	9.0	284

**Table 2:** Glass-transition temperatures of NOWPU films.

Sample	*T_gs_* (°C)	*T_gh_* (°C)	*ΔT_g_* (°C)
1,5-Dihydroxy naphthalene	−37.7	97.6	135.3
NOWPU-0	−44.8	79.9	124.7
NOWPU-0.5	−34.6	99.3	133.9
NOWPU-1.0	−40.7	99.8	140.5

**Table 3:** Maximum initial decomposition temperature (*T*_0_), maximum temperature (*T_max_*), and maximum decomposition speed (*V_max_*) of NOWPU films.

Sample	1st Stage			2nd Stage			3rd Stage			4th Stage			*T_com_* (°C)
	*T*_0_ (°C)	*V_max_* (%/min)	*T_max_* (°C)	*T*_0_ (°C)	*V_max_* (%/min)	*T_max_* (°C)	*T*_0_ (°C)	*V_max_* (%/min)	*T_max_* (°C)	*T*_0_ (°C)	*V_max_* (%/min)	*T_max_* (°C)	
1,5-Dihydroxy naphthalene	141	0.0304	248	-	-	-	-	-	-	-	-	-	274
NOWPU-0	180	0.0019	230	245	0.0039	309	324	0.0123	351	391	0.0040	440	470
NOWPU-0.5	174	0.0008	227	238	0.0049	315	318	0.0158	343	408	0.0026	427	500
NOWPU-1.0	176	0.0012	231	252	0.0072	324	343	0.0126	361	409	0.0047	456	500

**Table 4:** Limiting oxygen index (LOI) value, carbon residue yield and UL-94 result of NOWPU-0.5 and NOWPU-1.0.

Sample	LOI Value (%)	Carbon Residue Yield (%)	UL-94 Result
NOWPU-0.5	29.4	4.2	V-0
NOWPU-1.0	31.2	5.9	V-0

**Table 5:** Pencil hardness, transmittance and solvent resistance of NOWPU-0.5 and NOWPU-1.0.

Sample	Pencil Hardness	Transmittance (%)				Solvent Resistance
		UV 200 nm	UV 400 nm	Visible Light 800 nm	Natural Light	
NOWPU-0.5	H	22.2	78.3	82.7	91.2	Pass
NOWPU-1.0	2H	0.1	90.0	95.5	100.0	Pass

## References

[1] Chung Y.C., Yang K., Choi J.W., Chun B.C. (2014). Characterisation and application of polyurethane copolymers grafted with photoluminescent dyes. Color. Technol..

[2] Chung Y.C., Choi J.W., Lee H.L., Chun B.C. (2014). The characterization and effects of the alizarin series dyes grafted to polyurethane copolymers on the photoluminescence and low temperature flexibility. Fibers Polym..

[3] Guo L., Wang L., Huang S., Qu J. (2017). Synthesis and properties of novel water-dispersible polyisocyanates. J. Appl. Polym. Sci..

[4] Ge Z., Luo Y. (2013). Synthesis and characterization of siloxane-modified two-component waterborne polyurethane. Prog. Org. Coat..

[5] Wu G., Li J., Chai C., Ge Z., Lin J., Luo Y. (2015). Synthesis and characterization of novel post-chain extension flame retardant waterborne polyurethane. RSC Adv..

[6] Alcón M.J., Ribera G., Galià M., Cádiz V. (2005). Advanced flame-retardant epoxy resins from phosphorus-containing diol. J. Polym. Sci. Part A Polym. Chem..

[7] Chagnon L., Arnold G., Giljean S., Brogly M. (2013). Elastic recovery and creep properties of waterborne two-component polyurethanes investigated by micro-indentation. Prog. Org. Coat..

[8] Yin X., Luo Y., Zhang J. (2017). Synthesis and Characterization of Halogen-Free Flame Retardant Two-Component Waterborne Polyurethane by Different Modification. Ind. Eng. Chem. Res..

[9] Qu J., Tu W., Chen H. (2002). Synthesis and Characterization of Two Component Waterborne Polyurethane Paints. J. Chem. Eng. Chin. Univ..

[10] Park J., Lee J., Park T. (1996). Improved interfacial shear strength and durability of single carbon fiber reinforced isotactic polypropylene composites using water-dispersible graft copolymer as a coupling agent. Polym. Compos..

[11] Wu G.M., Chen J., Huo S.P., Liu G.F., Kong Z.W. (2014). Thermoset nanocomposites from two-component waterborne polyurethanes and cellulose whiskers. Carbohydr. Polym..

[12] Luo Y., Yin X., Chai C., Ge Z. (2017). A Kind of Mixed Hydrophilic Polyisocyanate Curing Agent and its Preparation Method. CN Patent.

[13] Hu X., Zhang X., Liu J., Dai J. (2014). Synthesis, characterization and fluorescence performance of a waterborne polyurethane-based fluorescent dye 4-amino-N-cyclohexyl-1,8-naphthalimide, WPU-ACN. Polym. Int..

[14] Hu X., Zhang X., Liu J., Dai J. (2013). Synthesis, characterization and fluorescence performance of a waterborne polyurethane-based polymeric dye. J. Lumin..

[15] Kim B.K., Lee J.C. (1996). Polyurethane ionomer dispersions from poly(neopertylene phthalate) glycol and isophorone diisocyanate. Polymer.

[16] Hu X.H., Zhang X.Y., Dai J.B. (2011). Synthesis and characterization of a novel waterborne stilbene-based polyurethane fluorescent brightener. Chin. Chem. Lett..

[17] Hu X., Zhang X., Liu J. (2015). Synthesis and Optical Performances of a Waterborne Polyurethane-Based Polymeric Dye. Int. J. Polym. Sci..

[18] Sheng Li W., Tie Jun S., Li Yuan Z. (2016). Latex co-coagulation approach to fabrication of polyurethane-graphene nanocomposites with improved electrical conductivity, thermal conductivity, and barrier property. J. Appl. Polym. Sci..

[19] Zhou C., Xie T., Zhou R., Trindle C.O., Tikman Y., Zhang X., Zhang G. (2015). Waterborne Polyurethanes with Tunable Fluorescence and Room-Temperature Phosphorescence. ACS Appl. Mater. Interfaces.

[20] Cho B.S., Kim J.S., Lee J.M., Kweon J.O., Noh S.T. (2014). Synthesis and characterization of poly(ferrocenyl glycidyl ether)-1,2-butylene oxide copolymers. Macromol. Res..

[21] Aguirresarobe R.H., Martin L., Fernandez-Berridi M.J., Irusta L. (2017). Autonomic healable waterborne organic-inorganic polyurethane hybrids based on aromatic disulfide moieties. Express Polym. Lett..

[22] Gallagher S. (2003). Synthesis and Characterization of Phosphonate-Containing Polysiloxanes. J. Polym. Sci. Part A Polym. Chem..

[23] Gu L., Luo Y. (2015). Flame Retardancy and Thermal Decomposition of Phosphorus-Containing Waterborne Polyurethanes Modified by Halogen-Free Flame Retardants. Ind. Eng. Chem. Res..

[24] Wu G., Li J., Luo Y. (2016). Flame retardancy and thermal degradation mechanism of a novel post-chain extension flame retardant waterborne polyurethane. Polym. Degrad. Stab..

[25] Zhang P., Tian S., Fan H., Chen Y., Yan J. (2015). Flame retardancy and hydrolysis resistance of waterborne polyurethane bearing organophosphate moieties lateral chain. Prog. Org. Coat..

[26] Zhang P., He Y., Tian S., Fan H., Chen Y., Yan J. (2017). Flame retardancy, mechanical, and thermal properties of waterborne polyurethane conjugated with a novel phosphorous-nitrogen intumescent flame retardant. Polym. Compos..

[27] Yu F., Cao L., Meng Z., Lin N., Liu X. (2016). Crosslinked waterborne polyurethane with high waterproof performance. Polym. Chem..

